# Development of a Novel Indirect ELISA for the Serological Diagnosis of African Swine Fever Using p11.5 Protein as a Target Antigen

**DOI:** 10.3390/pathogens12060774

**Published:** 2023-05-29

**Authors:** Mizuki Watanabe, Tomoya Kitamura, Koji Nagata, Mitsutaka Ikezawa, Ken-ichiro Kameyama, Kentaro Masujin, Takehiro Kokuho

**Affiliations:** 1 Nippon Institute for Biological Science, Tokyo 198-0024, Japan; 2 Division of Transboundary Animal Disease Research, National Institute of Animal Health (NIAH), National Agriculture and Food Research Organization (NARO), Tokyo 187-0022, Japan; 3 Department of Applied Biological Chemistry, Graduate School of Agricultural and Life Sciences, The University of Tokyo, Tokyo 113-8657, Japan

**Keywords:** African swine fever virus (ASFV), A137R, p11.5, serological ELISA

## Abstract

African swine fever is a hemorrhagic viral disease with a mortality rate of nearly 100% in pigs. Hence, it is classified as a notifiable disease by the World Organization for Animal Health. Because no field-available vaccine exists, African swine fever virus (ASFV) control and eradication solely depend on good farm biosecurity management and rapid and accurate diagnosis. In this study, we developed a new indirect serological enzyme-linked immunosorbent assay (ELISA) using recombinant p11.5 protein from ASFV as a solid-phase target antigen. The cutoffs were determined by receiver operating curve analysis performed with serum samples obtained from naïve and infected pigs. Based on the results of a commercially available serological ELISA, the relative sensitivity and specificity of our assay were 93.4% and 94.4% (N = 166; area under the curve = 0.991; 95% confidence interval = 0.982–0.999), respectively. Furthermore, to compare the performance of the serological ELISAs, we conducted the assays on a panel of sera collected from pigs and boars experimentally infected with different ASFV isolates. The results indicated the greater sensitivity of the newly developed assay and its ability to detect anti-ASFV antibodies earlier after virus inoculation.

## 1. Introduction

African swine fever (ASF) is a hemorrhagic viral disease with a maximum mortality rate of 100% in domestic pigs. Therefore, it is listed as a notifiable disease by the World Organization for Animal Health. The etiological agent, African swine fever virus (ASFV), is a large double-stranded DNA virus with icosahedral morphology [1,2].

ASF was first reported by Montgomery in Kenya in 1921 [3]. After a long period of circulation in Africa the virus suddenly spread to Europe and South America in 1957 and 1960, respectively [4]. In 2007, ASF emerged in Georgia and then spread to Armenia, Azerbaijan, and other Caucasian countries [5].

From 2014 to 2018, several European countries including Estonia, Lithuania, Latvia, Poland, Czech Republic, Bulgaria, Belgium, Romania, and Hungary experienced severe outbreaks of ASF. Since then, the disease has gradually spread to additional countries such as Germany, North Macedonia, and Italy. In Asia, ASF was first confirmed in China in 2018, and has since spread to nearly all neighboring countries with the exception of Japan and Chinese Taipei (as of April 2023). Moreover, ASF emerged in Papua New Guinea in Oceania in 2020, and in 2021, it affected the Dominican Republic and Haiti in Central America. As a result, the disease has had a significant socio-economic impact on global pork industries and poses threats to biosafety, biosecurity, and the sustainable supply of food for human consumption [4,6,7].

There is currently a high demand for effective vaccines against ASF, and research and development efforts are underway worldwide. Among these, live attenuated vaccines with genetic mutations or deletions in genes such as 9GL, EP402R (CD2v), DP148R, I177L, I226R, A137R, and E184L, as well as mutations in MGF and LVR gene loci, have been published. Additionally, recombinant viral vector vaccines such as rAd5 + MVA have been designed to express multiple antigenic proteins (B602L, B636L, CP204L, E183L, E199L, EP153R, F317L, and MGF505-5R), either individually or in combination [6,7]. However, despite these advancements, a vaccine that fully meets both safety and efficacy requirements has not yet become available. Therefore, its control and eradication solely depend on the implementation of appropriate biosecurity measures and rapid detection of infected animals [8].

Domestic pigs (*Sus scrofa domesticus*) are affected by ASFV, but manifestations of the disease can vary from highly lethal hemorrhagic, acute disease to subclinical illness depending on the virus strain [9,10]. By contrast, African wild suids such as warthogs (*Phacochoerus aethiopicus*) and bush pigs (*Potamochoerus larvatus*) exhibit milder symptoms or remain asymptomatic during infection. Wild boars (*Sus scrofa*) are also highly susceptible to ASFV, presenting symptoms similar to those of domestic pigs. Thus, they are major vectors of disease spread in many countries without vector ticks [11].

The rapid detection of ASFV-infected animals is imperative in outbreak areas. In cases of infestations by less virulent strains of ASFV or long-term persistence of the virus in endemic areas, serological diagnosis and surveillance are highly demanded. Serologic surveillance aims to detect antibodies against ASFV. Positive ASFV antibody test results can indicate ongoing or previous outbreaks. This is because some animals may recover and remain seropositive for a significant period, possibly life. This could include carrier animals [8].

This study aimed to develop a novel indirect serological enzyme-linked immunosorbent assay (ELISA) to detect immunological responses to avirulent or less virulent ASFVs with higher sensitivity than currently available commercial assays. In our preliminary experiments, to identify an appropriate antigen for serological ELISA, mouse monoclonal antibodies (mAbs) were generated against the pathogenic genotype II strain AQS-C-1-22 (AQS) [12], and the antigenic proteins were identified by screening using a panel of expression vectors encoding a complete set of 192 predicted open-reading frames of Georgia 2007/1, a highly pathogenic genotype II strain. p11.5, the protein encoded by the A137R gene, was identified as a major viral protein with particular antigenic activity.

Hence, we examined the suitability of this molecule as a target antigen in a solid-phase indirect ELISA for the serological detection of ASFV-specific antibodies in infected animals and established a sensitive serological assay system.

## 2. Materials and Methods

### 2.1. p11.5 Recombinant DNA Construction

**Figure 1:**
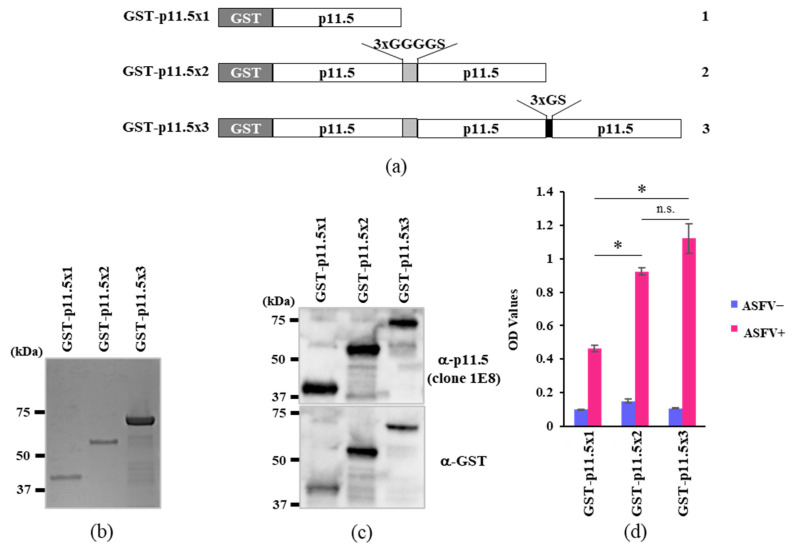
p11.5 antigen preparation and analysis. (**a**) Schematic presentation of the inserted sequences in the pCold-GST plasmids. Two different linkers, namely 3×GGGGS and 3×GS, are also shown. A GST tag was added to the N-terminal domain for purification and identification. Line 1, GST-p11.5×1; line 2, GST-p11.5×2; line 3, GST-p11.5×3. (**b**,**c**) GST-p11.5×1, GST-p11.5×2, and GST-p11.5×3 fusion proteins were expressed in *E. coli* BL21, solubilized, and purified on a GSTrap FF column. Half of the eluted proteins were analyzed by electrophoresis in a denaturing gel and stained with Coomassie brilliant blue G-250 (**b**), and the remaining proteins were analyzed by electrophoresis in a denaturing gel and immunoblotted with the indicated antibodies (**c**). The image is representative of three independent experiments. α, anti-. (**d**) Comparison of the OD_450_ values of ELISA using three different proteins as the target antigen. ASFV+, ASFV-positive serum samples (from pigs infected with ASFV OUR T88/3 at 21 dpi); ASFV−, ASFV-negative serum samples (from uninfected pigs). The antigen proteins were coated at a concentration of 5 μg/mL. The serum samples and secondary antibody were used at dilutions of 1:20 and 1:5000, respectively. Each data point is indicated as the mean ± standard error of three independent experiments. *, *p* < 0.01 (Tukey’s test); n.s., not significant.

The glutathione S-transferase (GST) tag sequence was inserted into pCold I DNA (Takara Bio Inc., Shiga, Japan) between the *Xho*I and *Eco*RI sites to create pCold-GST-DNA. The p11.5/A137R gene of ASFV Georgia 2007/1 strain (accession No. NC_044959.2 at DDBJ/EMBL/GenBank) was amplified using forward (5′-CCAGGGGCCCGAATTCATGGAAGCAGTTCTTACCAAAC-3′) and reverse primers (5′-TAGACTGCAGGTCGACTTAGCCTTCTTTGATATTCATC-3′). Three different expression plasmids featuring insertions of single, double, and triple p11.5 domain-encoding sequences were prepared by modifying the bacterial expression plasmid pCold-GST using the NEBuilder HiFi DNA assembly cloning kit (New England Biolabs, Ipswich, MA, USA). Briefly, linearized pCold-GST plasmid DNA was ligated to PCR-amplified fragments of p11.5 or to p11.5 with a 3×GGGGS linker sequence at the 3′-end in combination to yield GST-p11.5×1 and GST-p11.5×2 expression vectors, respectively. Similarly, another vector encoding three repetitive sequences of p11.5 with 3×GGGGS and 3×GS linker sequences (GST-p11.5×3) was prepared. The schematic diagram of the inserted sequences in the pCold-GST plasmids are indicated in Figure 1a. These plasmids were then introduced into *Escherichia coli* DH5α (Takara Bio) and incubated overnight at 37 °C on ampicillin-loaded agar plates. We confirmed the inserted sequences by Sanger sequencing (Applied Biosystems 3500 or Applied Biosystems SeqStudio Genetic Analyzer, Thermo Fisher Scientific Inc., Waltham, MA, USA).

### 2.2. Recombinant Protein Generation

Synthetic recombinant plasmids were introduced into *E. coli* BL21 (Takara Bio). Transformed BL21 cells were cultivated with a small amount of 2×YT broth (containing 50 μg/mL ampicillin) in a shaker at 200 rpm and 37 °C and then inoculated into a large bottle of 2×YT at a dilution of 1:100. *E. coli* was grown at 160 rpm and 19 °C when the optical density at 600 nm (OD_600_) reached 0.4–0.5. Then, 300 μM isopropyl-β-d-thiogalactopyranoside (Nacalai Tesque) was added to induce protein synthesis with overnight incubation at 160 rpm and 19 °C. Following centrifugation, bacteria were collected and suspended in lysis buffer (1 M NaCl, 50 mM Tris pH 8.0, 1% Triton X) containing protease inhibitor cocktails (Merck KGaA, Darmstadt, Germany) and sonicated. After centrifugation at 15,000 rpm for 10 min, the supernatants were collected and filtered through a 0.45 μm syringe filter, and the proteins were purified on a GSTrap FF column using an AKTA start liquid chromatography system (Cytiva, Tokyo, Japan) following standard protocols. Eluted proteins were fractionized by sodium dodecyl sulfate-polyacrylamide gel electrophoresis (SDS-PAGE) and stained for visualization with Coomassie brilliant blue G-250 (Bio-Safe CBB G-250 Stain, Nacalai Tesque).

### 2.3. Antibodies

Anti-p11.5 antibody (clone 1E8, IgG2b, κ) is a mAb against ASFV p11.5 previously generated in our laboratory. Goat polyclonal antibody against GST was purchased from Cytiva. A horseradish peroxidase (HRP)-conjugated antibody for indirect ELISA, namely Peroxidase AffiniPure Goat Anti-Swine IgG (H + L), was purchased from Jackson ImmunoResearch Inc. (West Grove, PA, USA). Goat anti-Mouse IgG (H + L) Cross-Adsorbed Secondary Antibody, Alexa Fluor 488 (Thermo Fisher Scientific Inc.) was obtained as a secondary antibody for immunofluorescence staining.

### 2.4. Serum Samples

#### 2.4.1. Animal Experiment and Virus Strains

Animal experiments were performed in compliance with the regulations outlined in the Guide for the Care and Use of Laboratory Animals of the National Institute of Animal Health (NIAH), National Agriculture and Food Research Organization (NARO), Guidelines for Proper Conduct of Animal Experiments of the Science Council of Japan [13], and ARRIVE guidelines [14]. The animal study was reviewed and approved by the Institutional Animal Care and Use Committee at NIAH, NARO (approval numbers: 21-023, 21-052, 22-026, 22-058, 22-059). During the study period, all pigs received weaning/growing diets, and they had free access to water. The animals were observed daily for clinical signs and/or welfare disturbances. Every effort was made to minimize animal distress and reduce the number of animals used. All pigs were checked for the absence of ASFV by quantitative PCR [15,16,17] after transduction and for the absence of anti-ASFV antibodies using an indirect ELISA kit (ID Screen African Swine Fever Indirect ELISA, IDvet, Grabels, France) [17]. Pigs were then randomly divided into groups. The virus was inoculated intramuscularly into all pigs. Clinical signs and body temperature were monitored daily, and blood samples were obtained at regular intervals. All pigs were then necropsied before the end of the experimental period. Euthanasia was considered a humane endpoint when pigs exhibited significant depression, and it was justified for welfare reasons. For each animal experiment, LWD pigs and wild boars were used. Five infection tests in animals were conducted using ASFV genotypes I, II, and X.

All positive serum samples (N = 76) were obtained from pigs experimentally infected with ASFV. OUR T88/3 (genotype I) [18], Lisbon60V (genotype I) [19], and Kenya05-Tk1 (genotype X) were obtained from the OIE reference laboratory for ASF (Universidad Complutense de Madrid, Spain). AQS-C-1-22 (AQS, genotype II) was described previously [16]. An attenuated AQS strain and Armenia 2007 (Arm07, genotype II) ΔMGF [17] were cloned in our previous studies. Details of the virus inoculation protocol are presented in Supplementary Table S1. Pig serum samples were obtained at regular intervals after inoculation. Anti-ASFV antibody positivity was determined by IDvet indirect ELISA. For negative control serum samples, we collected serum samples from experimental pigs before inoculation (N = 60).

#### 2.4.2. Serum Samples from the Field

ASFV-negative serum samples were collected from healthy pigs (N = 30) from commercial pig farms in Japan.

### 2.5. Immunoblotting

Western blotting was performed to confirm the expression of purified GST-tagged p11.5×1, p11.5×2, and p11.5×3 recombinant proteins by transformed bacteria. The bacterial lysates containing recombinant proteins were heated at 100 °C for 5 min in SDS sample buffer (Sample Buffer Solution with Reducing Reagent (6×) for SDS-PAGE; Nacalai Tesque), subjected to electrophoresis in denaturing gels (Mini-PROTEAN TGX Gels 4%–20%; Bio-Rad Laboratories, Inc., Hercules, CA, USA), and transferred to PVDF membranes. The membranes were blocked with Bullet Blocking One for Western Blotting (Nacalai Tesque) for 20 min and reacted with the indicated antibodies for at least 1 h at room temperature or overnight at 4 °C. These membranes were then reacted with secondary antibody conjugated with peroxidase (Jackson) and visualized using Chemi-Lumi One L or Chemi-Lumi One Super (Nacalai Tesque) with the chemiluminescence imaging system (ChemiDoc Touch; Bio-Rad Laboratories).

### 2.6. p11.5-Indirect ELISA

#### 2.6.1. Procedure for p11.5-Indirect ELISA

ELISA plates coated with recombinant proteins diluted in carbonate buffer (34 mM Na_2_CO_3_, 100 mM NaHCO_3_, pH 9.5) were incubated at 4 °C overnight or at room temperature for 2 h. Pig serum samples were diluted in ChonBlock Blocking/Sample Dilution Buffer (Chondrex, Inc., Woodinville, WA, USA) and added to each well of the plate after removing the coating antigen. Following incubation at 37 °C for 1 h, the plates were washed three times with 0.1% Tween in PBS (*v*/*v*, PBST), and Peroxidase AffiniPure Goat Anti-Swine IgG (H + L) at an indicated dilution was added to each well. After incubating at 37 °C for 1 h following three washes with PBST, 50 μL of the chromogenic substrate solution (TMB, Sera Care, Milford, MA, USA) was added. Color reaction was developed for 5 min and then stopped by adding 50 μL of 3 M sulfuric acid. OD_450_ of the reactions was measured using a Nivo ELISA plate reader (PerkinElmer Co., Ltd., Waltham, MA, USA). Antigen and antibody concentrations were optimized via checkerboard titration. Antigens were diluted to concentrations of 0.5–20 μg/mL, pig serum samples were diluted to 1:10–1:500, and HRP anti-swine IgG was diluted to 1:1000–1:10,000 to identify the optimal conditions. The optimal conditions were those for which OD_450_ of the positive serum samples exceeded 1, and the OD450 ratio (P/N value) of positive to negative serum samples was highest. Negative serum samples from 90 pigs in commercial pig farms and experimental pigs in the negative control groups in animal experiments and 76 positive serum samples from experimentally infected pigs confirmed positive for ASFV by IDvet indirect ELISA was used to calculate the cutoffs for the newly developed indirect ELISA. Cutoffs were indicated by OD_450_ of 0.386.

#### 2.6.2. Reproducibility of p11.5-Indirect ELISA

The intra- and inter-assay variations of the results of p11.5-indirect ELISA were evaluated using the coefficient of variation (CV). Five negative and positive serum samples each were randomly selected, tested in three replicates in one batch to evaluate intra-assay variation, and analyzed by three independent assays to evaluate inter-assay variation.

#### 2.6.3. Comparison of p11.5-Indirect ELISA and IDvet Indirect ELISA

In total, 166 pig serum samples were tested in duplicate by p11.5-indirect ELISA and IDvet indirect ELISA. The performance of the assays was evaluated by relative sensitivity [(true positive/true positive + false positive) × 100%] and relative specificity [(true negative/true negative + false positive) × 100%].

### 2.7. Statistical Analysis

We used EZR (The R Foundation for Statistical Computing, Vienna, Austria), a modified version of R commander that adds statistical functions commonly used in biostatistics, to draw receiver operating characteristic (ROC) curves [20]. For multiple comparisons, we used Tukey’s test. Significance was indicated by *p* < 0.05.

## 3. Results

### 3.1. Expression of p11.5 Recombinant Proteins

The A137R gene of the highly virulent genotype II ASFV isolate AQS was expressed as a GST-tagged protein in *E. coli* BL21. The nucleotide sequence of the A137R gene of AQS was 100% identical to that of Georgia 2007/1, which was considered to be the original strain of the present global epidemic. As shown in Figure 1a, GST-p11.5×2 and GST-p11.5×3 are GST-tagged proteins carrying two and three domains of p11.5 with a linker peptide insertion (3×GGGGS/3×GS), respectively. On SDS-PAGE, three major bands were observed at approximately 43, 59, and 75 kDa, respectively (Figure 1b). To confirm the expression of the expected proteins, Western blotting of the same gel was performed with anti-p11.5 mAb (clone 1E8) and anti-GST antibody (Figure 1c).

### 3.2. Assessment of the Recombinant Proteins as an Indirect ELISA Target Antigen

We compared the reactivity of recombinant p11.5 proteins in different forms in a solid-phase indirect ELISA format. Recombinant GST-p11.5×1, GST-p11.5×2, and GST-p11.5×3 were used for coating an assay plate at a concentration of 5 μg/mL, and the OD_450_ values of uninfected pig sera (negative) and pig sera infected with the avirulent strain OUR T88/3 (genotype I) was determined by colorimetric ELISA. The highest OD_450_ was achieved when recombinant GST-p11.5×3 was used as a coating antigen (Figure 1d). Hence, we selected recombinant GST-p11.5×3 as the target antigen of indirect ELISA (were in termed “p11.5-indirect ELISA”).

### 3.3. Optimization of the Working Conditions of p11.5-Indirect ELISA

**Figure 2:**
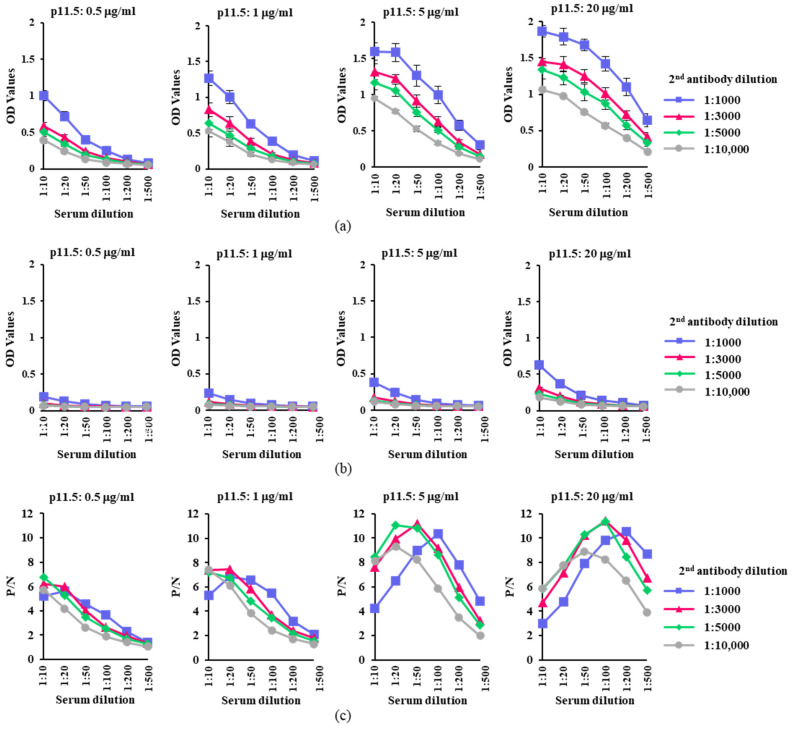
Determination of an appropriate amount of p11.5 protein for coating and optimal concentrations for test samples and the secondary antibody. (**a**,**b**) The p11.5 antigen at concentrations of 0.5, 1, 5, and 20 μg/mL; positive (OUR T88/3 infected pig sera) (**a**) or negative sera (normal pig sera) (**b**) at dilutions of 1:10, 1:20, 1:50, 1:100, 1:200, and 1:500; and anti-swine secondary antibody at dilutions of 1:1000, 1:3000, 1:5000, and 1:10,000 were used. An optimal reaction was obtained with a secondary antibody dilution of 1:5000. Each data point represents the mean ± standard error of three independent experiments; (**c**) The P/N ratios were calculated.

Next, we attempted to optimize p11.5-indirect ELISA using different amounts of the coating antigen and different dilutions of the secondary antibody. Figure 2a,b shows OD_450_ for ASFV-positive and ASFV-negative sera under different assay conditions, respectively. The highest P/N ratios of the reactions were observed when the secondary antibody was used at dilutions of 1:3000 and 1:5000 (Figure 2c). Hence, the secondary antibody was fixed at a dilution of 1:5000 for further investigation. Finally, we used recombinant p11.5 at a concentration of 5 μg/mL to coat the wells of ELISA plates, and the test sera were applied at a dilution of 1:20 for the assay, as these conditions yielded higher OD_450_ values and the highest P/N ratio for ASFV-positive and ASFV-negative sera (Table 1).

### 3.4. Standardization and Determination of the Cutoffs in p11.5-Indirect ELISA

**Figure 3:**
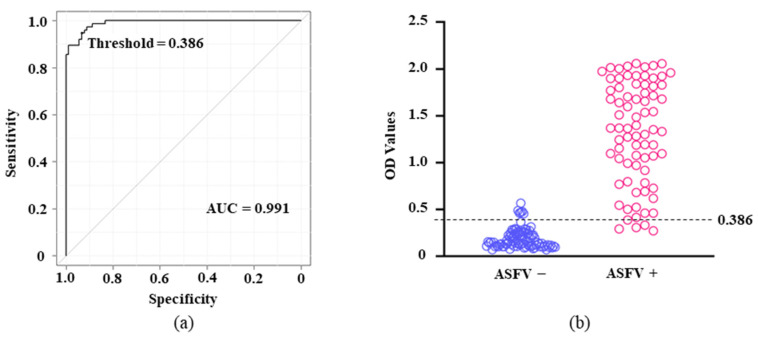
Standardization and determination of the cutoff for p11.5-indirect ELISA ROC analysis. In total, 166 pig sera (90 negative and 76 positive sera) were assayed by p11.5-indirect ELISA and IDvet indirect ELISA. (**a**) The AUC of p11.5-indirect ELISA was determined by ROC analysis (AUC = 0.991; 95% CI = 0.982–0.999). The threshold value (0.386) was calculated by EZR. (**b**) Each circle represents one serum sample of an individual pig. Values above and below the dashed line with an OD cutoff of 0.386 were considered positive and negative, respectively.

To determine the cutoff of the assay, 166 porcine sera (90 intact and 76 ASFV-infected) were tested in duplicate by p11.5-indirect ELISA using the optimized protocol. As shown in Figure 3a, the area under the ROC curve (AUC) with a threshold value of 0.386 was determined to be 0.991 [95% confidence interval (CI) = 0.982–0.999], indicating that the assay is “highly accurate.” Therefore, using this threshold (0.386) as the cutoff, we achieved a reliable serological assay with relative sensitivity of 93.4% (95% CI = 85.3–97.8) and relative specificity of 94.4% (95% CI = 87.5–98.2; Figure 3, Table 2).

### 3.5. Reproducibility of p11.5-Indirect ELISA

To evaluate the reproducibility of the developed assay, we examined the intra- and inter-assay CVs using uninfected and ASFV-infected porcine sera (one sample each from Groups 1–5; see Supplemental Table S1). The intra- and inter-assay CVs were both lower than 10%, clearly indicating the high repeatability of p11.5-indirect ELISA (Table 3).

### 3.6. Comparison of Serological ELISAs for Early Detection of Specific Antibodies in ASFV-Infected Animals

**Figure 4:**
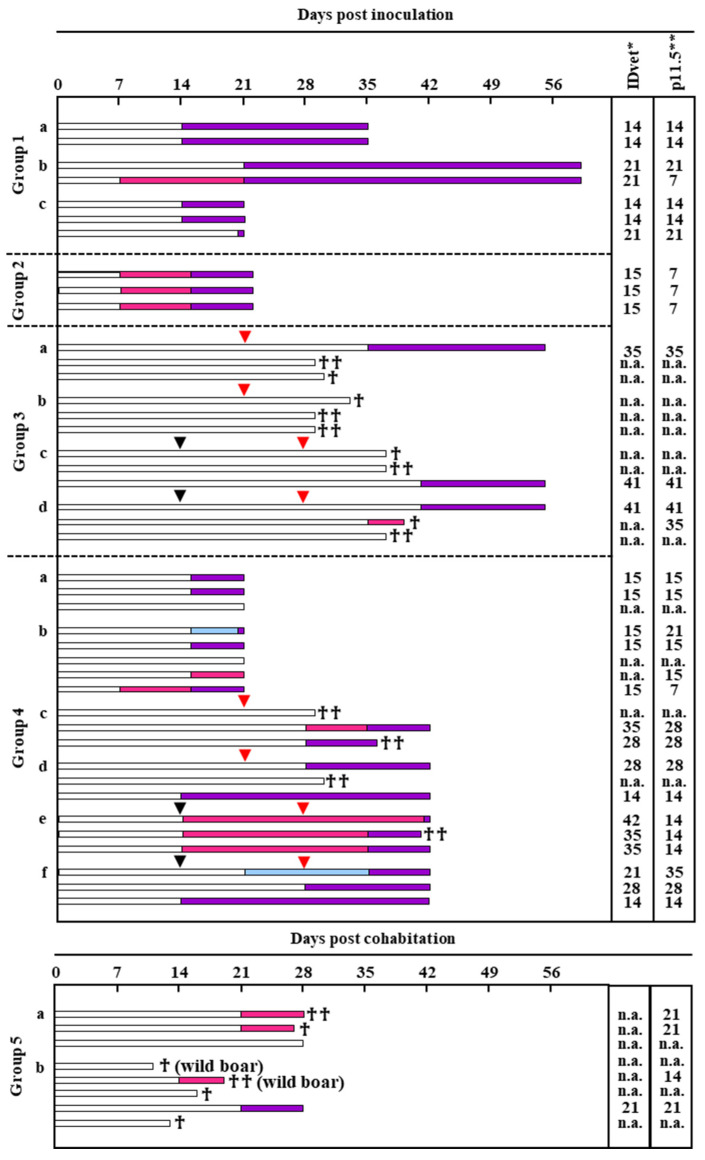
The time to the first detection of antibodies after ASFV inoculation in animals using a commercial ELISA kit or p11.5-indirect ELISA. Group 1, inoculated with OUR T88/3 (genotype I); Group 2, inoculated with Lisbon60V (genotype I); Group 3, inoculated with an attenuated AQS strain and wild-type AQS (genotype II); Group 4, inoculated with Arm07ΔMGF and wild-type AQS (genotype II); Group 5, cohabitation of a Kenya05/Tk-1 (genotype X)-infected pig and wild boar. Serum samples were collected from cohabitating animals. In the cohabitation study, ASFV-inoculated animals were excluded from this graph because they died at 9–13 dpi, and their serum samples were not collected. The exact dates of virus transmission from the inoculated individuals to the uninfected animals were unknown, and thus, the time was based on the dates of inoculation. An overview of virus inoculation is presented in Supplementary Table S1. In the graph, each bar represents one animal. White bars, the period during which the serum antibodies were negative in both indirect ELISAs; light blue bars, the period during which the serum antibodies appeared positive only in IDvet indirect ELISA; pink bars, the period during which the serum antibodies appeared positive only in the p11.5-indirect ELISA; purple bars, the period during which the serum antibodies appeared positive in both indirect ELISAs. The time until anti-ASFV antibodies were first detected by IDvet indirect ELISA * and p11.5-indirect ELISA ** is presented. Black arrow, additional inoculation of attenuated virus; red arrow, additional inoculation of virulent parental virus; n.a., no antibody. For deceased animals, the time to death ♰ or euthanasia because of human endpoints ♰♰ is indicated. All results represent pigs excluding the wild boar noted in brackets in Group 5b.

In the present study, we compared the performance of serological ELISAs in early detection of specific antibodies in pigs after the inoculation of genotypically varied attenuated ASFVs (Figure 4, Supplemental Table S1). In Group 1, four and three pigs were inoculated with the OUR T88/3 strain at doses of 1 × 10^6^ (Groups 1a and 1b) and 1 × 10^5^ TCID_50_/head (Group 1c), respectively. In Group 1, all pigs became seropositive by 21 days post-inoculation (dpi), whereas one pig was deemed positive at 7 dpi by p11.5-indirect ELISA but not by IDvet indirect ELISA. In Group 2, three pigs were inoculated with another attenuated genotype I ASFV strain (Lisbon60V) at a dose of 1 × 10^6^ TCID_50_/head. All pigs showed seroconversion at 7 dpi by p11.5-indirect ELISA and at 14 dpi by both ELISAs. In Group 3, three pigs each were inoculated with an attenuated derivative of AQS (unpublished) with one dose at 1 × 10^3^ TCID_50_/head (Group 3a), one dose at 1 × 10^5^ TCID_50_/head (Group 3b), two doses at 1 × 10^3^ TCID_50_/head (Group 3c), or two doses at 1 × 10^5^ TCID_50_/head (Group 3d). All of these pigs were then challenged with 1 × 10^2^ TCID_50_/head wild-type AQS at 21 (Groups 3a and 3b) or 28 dpi (Groups 3c and 3d). One pig each in Groups 3a, 3c, and 3d became positive at 35, 41, and 41 dpi, respectively, by both ELISAs, and seroconversion in one pig in Group 3d was only detected by p11.5-indirect ELISA at 35 dpi. In Group 4, three and five pigs (Groups 4a and 4b) were inoculated with another attenuated genotype II ASFV strain (Arm07ΔMGF [17]) at doses of 1 × 10^5^ and 1 × 10^7^ TCID_50_/head, respectively. Another three pigs each were inoculated with a single dose of the same virus at doses of 1 × 10^3^ (Group 4c) and 1 × 10^5^ TCID_50_/head (Group 4d), respectively. Additionally, three pigs each were inoculated twice with the same virus at doses of 1 × 10^3^ (Group 4e) and 1 × 10^5^ TCID_50_/head (Group 4f), respectively. Group 4c–f pigs were injected with 1 × 10^2^ TCID_50_/head wild-type AQS at 21 (4c and 4d) or 28 dpi (4e and 4f). One pig each in Groups 4b and 4f tested positive for the virus earlier by IDvet indirect ELISA (15 and 21 dpi, respectively) than by p11.5-indirect ELISA (21 and 35 dpi, respectively). Conversely, according to p11.5-indirect ELISA, six pigs tested positive at 7 and 15 dpi (Group 4b), 28 dpi (Group 4c), and 14 dpi (Group 4e). For the remaining 12 pigs in this group, similar results were obtained by both ELISAs.

In the last experimental group (Group 5), we conducted cohabitation studies among wild boars inoculated with a genotype X ASFV (Kenya05/Tk-1) and naïve animals. In Group 5a, one wild boar that was injected 1 × 10^1^ TCID_50_ of Kenya05/Tk-1 cohabitated with three susceptible naïve pigs on the day of injection. Although the exact date of transmission was unclear, two of three pigs showed seroconversion after 21 days of cohabitation by p11.5-indirect ELISA but not by IDvet indirect ELISA. Similarly, in Group 5b, two Kenya05/Tk-1–inoculated wild boars (1 × 10^3^ TCID_50_/head) cohabitated with two naïve wild boars and three naïve pigs on the day of inoculation. Among the cohabitating animals, one wild boar and one pig became seropositive after 14 and 21 days of cohabitation, respectively, when examined by p11.5-indirect ELISA (wild boar) or both ELISAs (pig). In Groups 5a and 5b, we did not examine the serum samples of virus inoculated wild boars; hence, we omitted the data of those animals. Table 4 summarizes the results of all serological assays. For the detection of genotype I virus-specific antibodies in pigs (Groups 1 and 2), seroconversion was detected in all ASFV-inoculated pigs at later phases of infection, but p11.5-indirect ELISA allowed earlier antibody detection than IDvet indirect ELISA. Regarding the detection of genotype II virus-specific antibodies, although we found some inconsistent results in Arm07ΔMGF-inoculated pigs (Groups 4b and 4f), the time to detection was slightly faster for p11.5-indirect ELISA than for IDvet indirect ELISA. For the detection of genotype X virus-specific antibodies (Group 5), p11.5-indirect ELISA permitted more sensitive and earlier detection of humoral responses in ASFV-infected animals.

## 4. Discussion

Currently, ASFV vaccines remain under development [21]. Therefore, disease prevention largely focuses on the implementation of effective biosecurity measures and the rapid detection and removal of affected animals. Antibody detection is particularly informative for monitoring virus circulation in the field, and it will possibly be useful for assessing immunological responsiveness in vaccinated animals when reliable vaccines become available for practical use [8]. For serological disease diagnosis, different types of serological ELISAs employing different target antigens such as indirect ELISAs using a mixture of ASFV p72, p62, and p32 (ID Screen African Swine Fever Indirect ELISA) or p30 (SVANOVIR ASFV-Ab, INDICAL, Uppsala, Sweden), blocking ELISA using p72 (INGEZIM PPA COMPAC, Ingenasa, Madrid, Spain), and competitive ELISA using p32 (ID Screen African Swine Fever Competition) are commercially available worldwide.

In our preliminary studies of ASFV-specific mAbs prepared from mice immunized with highly virulent viruses, we found that most of the established hybridoma clones produced antibodies against limited types of viral (mainly structural) proteins such as p72 and p11.5. Previous reports illustrated that p11.5 is the most abundantly expressed viral protein of ASFV during the replication cycle in vitro [16,17]. This suggests that p11.5 could likely be an appropriate target antigen for serological diagnosis.

p11.5 is a structural protein of ASFV encoded by the A137R gene. Gladue et al. recently reported that deletion of the gene in the highly virulent ASFV Georgia2010 (genotype II) significantly reduced its virulence in pigs, suggesting that “A137R-deficient Georgia2010” might be a vaccine candidate for ASFV [22]. Although the localization of p11.5 in a viral particle remains to be elucidated, it should be noted that both nucleotide and deduced amino acid sequences of the A137R gene are well conserved among isolates of various genotypes. Combined with its strong antigenicity, we speculate that ASFV p11.5 is an ideal target in serological investigations of various ASFVs of different genotypes. In this study, we examined the ability of p11.5-indirect ELISA to detect antibodies against genotype I, II, and X viruses and demonstrated the apparent applicability that this assay to the serological diagnosis of genetically various ASFV strains.

The competence of p11.5-indirect ELISA in early detection of ASFV-specific antibodies in affected animals was also evaluated, and with a few exceptions, the assay detected ASFV-specific antibodies in the samples at earlier times after infection than IDvet indirect ELISA regardless of the genotype tested. In addition, the assay detected antibody responses in some samples obtained from pigs inoculated with genotype II and genotype X viruses, whereas no antibody response was detected by IDvet indirect ELISA. Furthermore, in naïve animals cohabitated with wild boars infected with Kenya05/Tk-1, one susceptible wild boar tested positive by p11.5-indirect ELISA but remained negative throughout the experimental period by IDvet indirect ELISA. These results supported the superior sensitivity of p11.5-indirect ELISA compared to the commercial kit.

This novel indirect ELISA facilitated early detection of specific antibodies in ASFV-infected animals, permitting the practical identification of chronically infected pigs and precise monitoring of immunological responsiveness to ASFV raised by vaccines in future.

## 5. Conclusions

In this study, we developed a new indirect ELISA for serological diagnosis of ASF using the ASFV-derived p11.5 protein as the target antigen. The performance of the new ELISA was similar to or better than that of a currently available and globally used commercial product. The developed assay could permit early and reliable detection of ASF-affected animals, and it has the potential to diagnose disease caused by various strains of ASFV of different genotypes. Therefore, p11.5-indirect ELISA is promising as a tool for the serological diagnosis and field surveillance of ASF and the assessment of immunization efficacy when ASF vaccines become available in the future.

## Figures and Tables

**Table 1:** Determination of the optimal dilutions of coating antigens and test samples.

Antigen Concentration		Serum Dilution					
		1:10	1:20	1:50	1:100	1:200	1:500
20 μg/mL	P	1.336	1.226	1.028	0.876	0.577	0.331
	N	0.230	0.159	0.100	0.077	0.068	0.058
	P/N	5.81	7.73	10.31	11.38	8.44	5.74
5 μg/mL	P	1.171	1.062	0.761	0.510	0.277	0.152
	N	0.139	0.096	0.070	0.059	0.054	0.053
	P/N	8.42	11.06	10.82	8.60	5.10	2.88
1 μg/mL	P	0.637	0.470	0.285	0.177	0.107	0.071
	N	0.089	0.070	0.059	0.051	0.049	0.046
	P/N	7.19	6.75	4.85	3.48	2.17	1.54
0.5 μg/mL	P	0.506	0.341	0.188	0.128	0.081	0.059
	N	0.075	0.064	0.054	0.050	0.048	0.047
	P/N	6.75	5.30	3.48	2.54	1.71	1.26

α-swine secondary antibody, 1:5000; P, OD450 of infected animals (OUR T88/3-infected pig sera); N, OD450 of uninfected animals (normal pig sera); P/N, P/N ratio. The optimized condition is denoted by a red box. Each well of the plates was coated with 5 μg/mL p11.5, and for testing samples, a dilution of 1:20 was used for further analysis.

**Table 2:** Comparison of p11.5-indirect ELISA and the commercial kits.

p11.5-Indirect ELISA	IDvet-Indirect ELISA		
	+	−	Total
+	71	5	76
−	5	85	90
Total	76	90	166

Relative sensitivity = 93.4% (71/76), relative specificity = 94.4% (85/90).

**Table 3:** Reproducibility of p11.5-indirect ELISA.

		Intra-Assay			Inter-Assay		
Samples		Mean OD Value	SD	CV%	Mean OD Value	SD	CV%
Negative	No.1	0.141	0.009	6.39	0.142	0.003	2.01
	No.2	0.107	0.002	1.58	0.103	0.009	8.7
	No.3	0.109	0.003	2.64	0.120	0.011	8.79
	No.4	0.084	0	0	0.098	0.002	2.09
	No.5	0.087	0.002	2.48	0.092	0.004	4.57
Positive	Group 1	1.440	0.045	3.09	1.440	0.028	1.95
	Group 2	1.390	0.068	4.86	1.630	0.09	5.54
	Group 3	0.919	0.081	8.81	0.908	0.024	2.59
	Group 4	1.430	0.073	5.06	1.460	0.081	5.55
	Group 5	0.665	0.014	2.09	0.780	0.068	8.75

The assay was conducted in three replicates for each sample in one assay to determine the intra-assay variation or performed three times separately to determine the inter-assay variation.

**Table 4:** Comparison of the performance of p11.5-indirect ELISA and commercial ELISA in the detection of specific antibodies in ASFV-infected animals.

	Genotype I		Genotype II		Genotype X
	Group 1	Group 2	Group 3	Group 4	Group 5
IDvet-indirect ELISA	7 (17)	3 (15)	3 (39)	15 (23.7)	1 (21)
p11.5-indirect ELISA	7 (15)	3 (7)	4 (38)	16 (19.1)	3 (21), 1 * (14)
Number of pigs used in the experiment.	7	3	12	20	6, 2 *

The number of animals that appeared positive for the antibodies by the end of the study is presented, and the number in parentheses indicates the mean time to a positive result after inoculation. When calculating the mean, samples that tested negative throughout the experimental period or until the death animals were excluded. *, a result obtained from a cohabitated wild boar.

## Data Availability

Not applicable.

## Supplementary Materials

The following supporting information can be downloaded at: https://www.mdpi.com/article/10.3390/pathogens12060774/s1, Table S1: Details of the ASFV inoculation protocol for pigs.
