# Supplementary material for: Development of a Novel Indirect ELISA for the Serological Diagnosis of African Swine Fever Using p11.5 Protein as a Target Antigen
Source: Pathogens. 2023 May 29;12(6):774. doi: 10.3390/pathogens12060774 (PMC10304200; doi:10.3390/pathogens12060774)
Supplement: Supplementary file 1 [file pathogens-12-00774-s001.zip › pathogens-2419392-supplementary.pptx]

## Slide 1
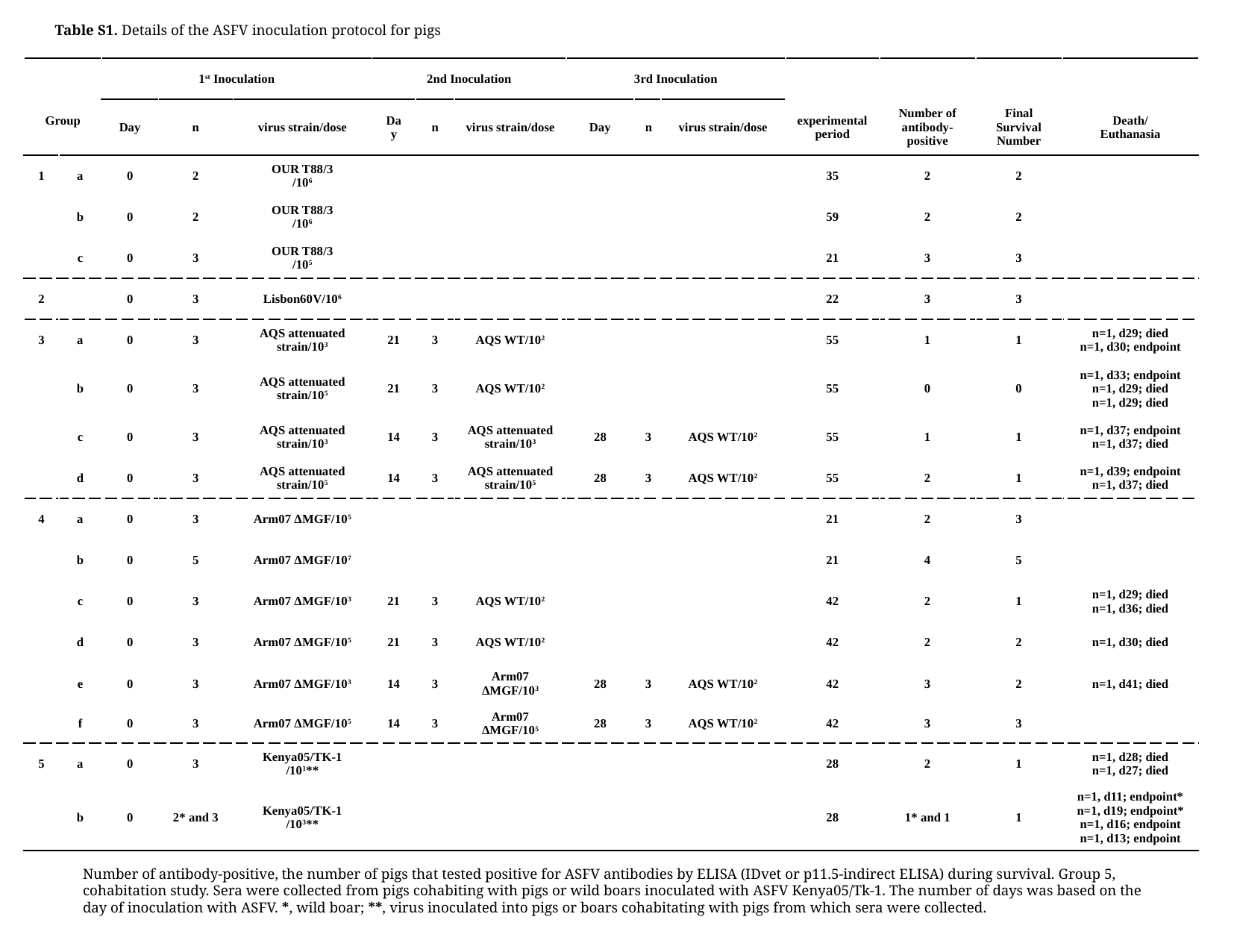

Table S1. Details of the ASFV inoculation protocol for pigs
| | | 1st Inoculation | | | 2nd Inoculation | | | 3rd Inoculation | | | | | | |
| --- | --- | --- | --- | --- | --- | --- | --- | --- | --- | --- | --- | --- | --- | --- |
| Group | | Day | n | virus strain/dose | Day | n | virus strain/dose | Day | n | virus strain/dose | experimental period | Number of antibody-positive | Final Survival Number | Death/ Euthanasia |
| 1 | a | 0 | 2 | OUR T88/3 /106 | | | | | | | 35 | 2 | 2 | |
| | b | 0 | 2 | OUR T88/3 /106 | | | | | | | 59 | 2 | 2 | |
| | c | 0 | 3 | OUR T88/3 /105 | | | | | | | 21 | 3 | 3 | |
| 2 | | 0 | 3 | Lisbon60V/106 | | | | | | | 22 | 3 | 3 | |
| 3 | a | 0 | 3 | AQS attenuated strain/103 | 21 | 3 | AQS WT/102 | | | | 55 | 1 | 1 | n=1, d29; died n=1, d30; endpoint |
| | b | 0 | 3 | AQS attenuated strain/105 | 21 | 3 | AQS WT/102 | | | | 55 | 0 | 0 | n=1, d33; endpoint n=1, d29; died n=1, d29; died |
| | c | 0 | 3 | AQS attenuated strain/103 | 14 | 3 | AQS attenuated strain/103 | 28 | 3 | AQS WT/102 | 55 | 1 | 1 | n=1, d37; endpoint n=1, d37; died |
| | d | 0 | 3 | AQS attenuated strain/105 | 14 | 3 | AQS attenuated strain/105 | 28 | 3 | AQS WT/102 | 55 | 2 | 1 | n=1, d39; endpoint n=1, d37; died |
| 4 | a | 0 | 3 | Arm07 ΔMGF/105 | | | | | | | 21 | 2 | 3 | |
| | b | 0 | 5 | Arm07 ΔMGF/107 | | | | | | | 21 | 4 | 5 | |
| | c | 0 | 3 | Arm07 ΔMGF/103 | 21 | 3 | AQS WT/102 | | | | 42 | 2 | 1 | n=1, d29; died n=1, d36; died |
| | d | 0 | 3 | Arm07 ΔMGF/105 | 21 | 3 | AQS WT/102 | | | | 42 | 2 | 2 | n=1, d30; died |
| | e | 0 | 3 | Arm07 ΔMGF/103 | 14 | 3 | Arm07 ΔMGF/103 | 28 | 3 | AQS WT/102 | 42 | 3 | 2 | n=1, d41; died |
| | f | 0 | 3 | Arm07 ΔMGF/105 | 14 | 3 | Arm07 ΔMGF/105 | 28 | 3 | AQS WT/102 | 42 | 3 | 3 | |
| 5 | a | 0 | 3 | Kenya05/TK-1 /101\*\* | | | | | | | 28 | 2 | 1 | n=1, d28; died n=1, d27; died |
| | b | 0 | 2\* and 3 | Kenya05/TK-1 /103\*\* | | | | | | | 28 | 1\* and 1 | 1 | n=1, d11; endpoint\* n=1, d19; endpoint\* n=1, d16; endpoint n=1, d13; endpoint |
Number of antibody-positive, the number of pigs that tested positive for ASFV antibodies by ELISA (IDvet or p11.5-indirect ELISA) during survival. Group 5, cohabitation study. Sera were collected from pigs cohabiting with pigs or wild boars inoculated with ASFV Kenya05/Tk-1. The number of days was based on the day of inoculation with ASFV. *, wild boar; **, virus inoculated into pigs or boars cohabitating with pigs from which sera were collected.
